# Parliament and the Profession

**Published:** 1919-11-08

**Authors:** 


### Parliament and the Profession.

### British Military Hospitals at Marseilles.

In answer to a question by Colonel Ward, Mr. Lambert stated that on October 16 there were at Marseilles one British General Hospital of 750 beds, and one Indian General Hospital of 400 beds.

### Maintenance of Voluntary Hospitals.

In answer to a question, Dr. Addison stated that he is aware of the increasing difficulties of many of the voluntary hospitals, and recognises that they affect the proper provision of various forms of medical services in hospitals and in centres or clinics and otherwise, but that he is not at present in a position to make a statement on the matter.

### United Service Massage League.

Sir Norton Griffiths asked the Financial Secretary to the War Office whether he will reconsider the recent decision that members of the United Service Massage League will receive no gratuity, and whether he would take into consideration the fact that these ladies signed on for six months and were under military discipline, therefore appearing to be entitled to the same treatment as that accorded to hospital workers. Mr. Forster said he was looking into the matter, but must not be understood to make any promise to alter the decision already announced.

### Influenza Prevention.

Dr. Addison stated that it is proposed that the Ministry of Health will issue a circular advising sanitary authorities and the public as to the preventive and other measures which should be taken this winter in regard to influenza.

### Quinine.

Mr. Spoor inquired of the President of the Board of Trade as to an alleged monopoly in quinine, for which 3s. 6d. per ounce is charged, as against 1s. before the war, and asked if he proposed to take any steps. Mr. Bridgeman replied that the whole question of the prices and supply of quinine sulphate is being investigated under the Profiteering Act.

